# Clopidogrel-loaded vascular grafts prepared using digital light processing 3D printing

**DOI:** 10.1007/s13346-023-01484-8

**Published:** 2023-12-05

**Authors:** Masoud Adhami, Camila J. Picco, Usanee Detamornrat, Qonita K. Anjani, Victoria A. Cornelius, Pamela Robles-Martinez, Andriana Margariti, Ryan F. Donnelly, Juan Domínguez-Robles, Eneko Larrañeta

**Affiliations:** 1 School of Pharmacy, Queen’s University Belfast, Belfast, BT9 7BL Northern Ireland UK; 2 Wellcome-Wolfson Institute for Experimental Medicine, Queen’s University Belfast, Belfast, BT9 7BL UK; 3 UCL School of Pharmacy, University College London, WC1N 1AX London, UK; 4 Department of Pharmacy and Pharmaceutical Technology, University of Seville, Seville, Spain

**Keywords:** 3D printing, Digital light processing, Vascular grafts, Clopidogrel

## Abstract

The leading cause of death worldwide and a significant factor in decreased quality of life are the cardiovascular diseases. Endovascular operations like angioplasty, stent placement, or atherectomy are often used in vascular surgery to either dilate a narrowed blood artery or remove a blockage. As an alternative, a vascular transplant may be utilised to replace or bypass a dysfunctional or blocked blood vessel. Despite the advancements in endovascular surgery and its popularisation over the past few decades, vascular bypass grafting remains prevalent and is considered the best option for patients in need of long-term revascularisation treatments. Consequently, the demand for synthetic vascular grafts composed of biocompatible materials persists. To address this need, biodegradable clopidogrel (CLOP)-loaded vascular grafts have been fabricated using the digital light processing (DLP) 3D printing technique. A mixture of polylactic acid-polyurethane acrylate (PLA-PUA), low molecular weight polycaprolactone (L-PCL), and CLOP was used to achieve the required mechanical and biological properties for vascular grafts. The 3D printing technology provides precise detail in terms of shape and size, which lead to the fabrication of customised vascular grafts. The fabricated vascular grafts were fully characterised using different techniques, and finally, the drug release was evaluated. Results suggested that the performed 3D-printed small-diameter vascular grafts containing the highest CLOP cargo (20% w/w) were able to provide a sustained drug release for up to 27 days. Furthermore, all the CLOP-loaded 3D-printed materials resulted in a substantial reduction of the platelet deposition across their surface compared to the blank materials containing no drug. Haemolysis percentage for all the 3D-printed samples was lower than 5%. Moreover, 3D-printed materials were able to provide a supportive environment for cellular attachment, viability, and growth. A substantial increase in cell growth was detected between the blank and drug-loaded grafts.

**Graphical Abstract:**

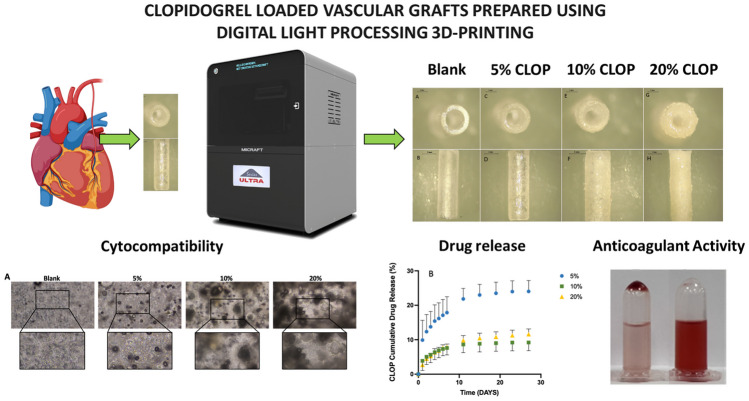

## Introduction

Diseases of the heart and blood vessels are collectively referred to as cardiovascular disease (CVD), which is the leading cause of death worldwide and a significant factor in decreased quality of life [1, 2]. More than 17.5 million people die every year from CVDs, and this number is expected to rise to 23.6 million by the year 2030 [3]. In the UK, CVDs are responsible for an average of 460 fatalities each day, or one death every 3 min [4]. Coronary artery disease (CAD) is the most prevalent form of CVDs. CAD is characterised by the gradual build-up of lipid-rich deposits inside the lumen of the arteries. Moreover, atherosclerosis may lead to several catastrophic consequences, the most serious of which are strokes and heart attacks [5]. Atherosclerosis is a major contributor to coronary heart disease (CHD), which causes the hardening of blood vessels and the formation of arterial thrombosis [6]. The number of affected arteries, severity, and location of the blockage all play a role in determining the best course of treatment for this condition. Stenting and peripheral and coronary bypass surgery have been among the most popular therapies for blocked blood arteries [7, 8].

One of the major complications following bypass surgery is the high risk of thrombosis formation. When it comes to its prevention, aspirin is often regarded as the antiplatelet medication of choice [9]. This suggestion is supported by recent meta-analyses, despite the fact that it is associated with an increased risk of bleeding due to the systemic effect of aspirin [10, 11]. Emerging research suggests that dual antithrombosis treatment with aspirin and clopidogrel or ticagrelor may be beneficial following coronary artery bypass graft surgery [12]. Even though the procedure is quite effective in the majority of patients, there is a concern over the availability of natural grafts for nearly 30% of those patients who require several grafts [13]. Consequently, the demand for synthetic vascular grafts composed of biocompatible materials persists [14, 15]. These synthetic vascular grafts have benefits in that they may be employed in many practical contexts due to their shape flexibility, length, and diameter [16, 17].

Recent research indicates that although synthetic grafts made of polyurethane, polyethylene terephthalate, and polytetrafluoroethylene are phenomenal in replacing large blood vessels, showing outstanding biocompatibility, low toxicity, and chemical stability, they are only marginally successful in replacing small-diameter blood vessels (< 6 mm internal diameter). The main associated complications are intima hyperplasia, thrombosis, infection, and delayed reendothelialisation [17, 18]. However, the large-diameter vascular grafts (> 8 mm internal diameter) have shown a high percentage of patency (90%) after the first year after implantation [19, 20]. Moreover, after the first year of implantation, patency rates for medium-diameter vascular grafts (internal diameter < 6 mm), such as carotid replacements, are slightly over 60% [21]. Accordingly, research is still being undertaken to determine the most effective method of manufacturing small-diameter vascular grafts in reconstructive surgery to overcome their limitations.

To mitigate these complications, numerous drugs can be incorporated for the manufacture of small-diameter vascular grafts with enhanced biological capabilities [15, 22–24]. For instance, drugs such as antibiotics have been widely employed in the development of cardiovascular grafts with anti-infective properties [15, 25]. Furthermore, the effectiveness of antithrombotic drugs like cilostazol, dipyridamole (DIP), acetylsalicylic acid, heparin, and cilostazol has been previously investigated as a preventive strategy against intimal hyperplasia and thrombosis [24, 26–28].

Three-dimensional (3D) printing technology sparked a revolution in the fields of pharmaceuticals, tissue engineering, and medical applications [29–31]. The immense potential of this technology is that the printed structures can feature solitary characteristics, such as the ability to load a wide variety of drug molecules, flexible dosing, and controlled release over time; as a result, they are suitable for use in personalised applications that cater to the clinical requirements of individual patients [32–34]. Digital Light Processing (DLP) 3D printers have the capability to flash a picture of a layer over the whole platform using a digital projector screen, curing every spot at once by the photo-polymerisation technique [35]. Indeed, small-diameter vascular grafts have already been fabricated with good printing resolution by using this 3D printing technology [36].

Biodegradable implants are made from polymers or block copolymers, both of which have the ability to be broken into smaller components which the body will then either excrete or absorb in due course [37–39]. In most cases, polymers such as poly(caprolactone) (PCL), polyglycerol sebacate (PGS), poly (lactic acid) (PLA), or poly (lactic-co-glycolic acid) (PLGA) are used in the manufacturing process [39]. Accordingly, these materials have been subjected to substantial research. Moreover, it is quite simple to alter their degradation kinetics in order to change the pace at which the drug is released. Because they are broken down in the body over time, it is not necessary to remove them once they have been implanted in the patient [40]. Furthermore, these materials may be used in the vat polymerisation technique to manufacture small-sized medical devices with high precision and accuracy. In the same vein, Xu et al. [41] developed an ocular drug delivery implant using the vat polymerisation technique.

The current study is intended to fabricate small-diameter vascular grafts using DLP 3D printing. The decision to use a DLP 3D printer was made in order to obtain greater resolution while also reducing the amount of time needed for production. The small-diameter vascular grafts developed in this study were composed of a polymeric mixture of polylactic acid-polyurethane acrylate (PLA-PUA) and low molecular weight PCL (L-PCL) containing different concentrations of CLOP. The small-diameter vascular grafts were extensively characterised, and the release of CLOP from this medical device was evaluated for 30 days. Moreover, the small-diameter vascular grafts were evaluated by exploring their antiplatelet effect, haemocompatibility, and cytocompatibility.

## Materials and method

### Materials

Polylactic acid-polyurethane acrylate (PLA-PUA) was purchased from Shenzhen Esun Industrial Co. Ltd. (Shenzhen, China). Low molecular weight PCL (L-PCL) (CAPA™ 2054, MW = 550 g/mol) was obtained from Ingevity UK Ltd. (Warrington, UK). Phenylbis (2,4,6–trimethyl benzoyl) phosphine oxide was purchased from Tokyo Chemical Industry UK Ltd. (Oxford, UK). (S)-( +)-clopidogrel sulfate (CLOP) was provided by Tokyo Chemical Industry UK Ltd. (Oxford, UK). Analytical grade isopropyl alcohol was obtained from MB Fibreglass (Newtownabbey, England). Glutaraldehyde 25% EM grade was purchased from Agar Scientific Ltd. (Essex, UK). Phosphate buffer saline (PBS) (pH 7.4) was obtained from VWR Chemicals (Ohio, USA). Reagent grade TWEEN® 80 was obtained from VWR Chemicals (Ohio, USA). HPLC-grade acetonitrile was purchased from Sigma-Aldrich (Basingstoke, England).

### 3D-printed vascular graft design and fabrication

The composition of the resin used for 3D printing was optimised. The optimum formulation to achieve a printable resin contained a mixture of PLA-PUA/L-PCL 65%/35% w/w and 1% w/w phenylbis (2,4,6-trimethyl benzoyl) phosphine oxide with respect to the weight of the PLA-PUA. The prepared mixture was combined with different concentrations of CLOP (5%, 10%, and 20% w/w) and mixed using the SpeedMixer™ DAC 150.1 FVZ-K (Hauschild GmbH & Co. KG, Westfalen, Germany) at 3500 rpm for 5 min before the 3D printing process. In brief, in order to get the best possible mixing results, the PLA-PUA, L-PCL, and phenylbis were initially mixed for 3 min at 3500 rpm in the SpeedMixer™ DAC 150.1 FVZ-K and then another 2 min after CLOP was added.

The vascular grafts with dimensions of 3.0 mm external diameter, 2.0 mm internal diameter, and 10.0 mm in length were designed using a computer-aided design (CAD) software and printed using a DLP 3D printer (MiiCraft 125 series) (Taiwan). The number of base layers was limited to one, and the first curing time was set at 50 s; this time was cut down to 10 s for the subsequent layers. Each layer was designed to be 100 µm thick. After the printing process was finished, the components were washed for 30 s with isopropyl alcohol (IPA) to remove any uncured resin from the surface of the printed material. The washed and dried vascular grafts were placed under UV light for 30 min to achieve their maximum potential level of strength and stability.

### Characterisation of 3D-printed vascular grafts

#### Microscopy study

The surface morphology of the 3D-printed vascular grafts was assessed using a Leica EZ4 D light microscope (Leica, Wetzlar, Germany) and scanning electron microscopy (SEM) (Hitachi TM3030; Tokyo, Japan).

#### Fourier transform infrared (FTIR) spectroscopy

The attenuated total reflectance (ATR) approach was used to record the Fourier transform infrared (FTIR) spectra of the pure CLOP and the 3D-printed vascular grafts using a Spectrum Two instrument (Perkin Elmer, Waltham, MA, USA). The spectra were collected with a resolution of 4 cm^−1^ and spanned the range of 4000 to 600 cm^−1^ at 64 scans.

#### Thermal analysis

Thermogravimetric (TGA) and differential scanning calorimetry (DSC) studies were used to assess the thermal properties of the pure CLOP and the 3D-printed vascular grafts. Degradation temperatures of 3D-printed products and pure CLOP were determined using TGA. A small portion of the pure drug and the grafts (between 3 and 10 mg) were utilised for this analysis. TGA was conducted by employing a Q50 Thermogravimetric analysis (TA instruments, Bellingham, WA, USA). Under a nitrogen flow rate of 50 mL/min, samples were heated from 20 to 500 °C at a rate of 10 °C/min. In addition, the drug’s potential for becoming an amorphous dispersion after combining with the polymer matrix was assessed using a Q20 differential scanning calorimeter (TA instruments, Bellingham, WA, USA). Similarly, a small portion of the pure drug and the grafts (between 3 and 10 mg) were utilised for this analysis. Under a nitrogen flow rate of 50 mL/min, scans were taken from 30 to 300 °C at a rate of 10 °C/min.

#### X-ray diffraction (XRD) analysis

X-ray diffraction (XRD) analysis was employed to assess the crystalline structure of CLOP in the 3D-printed vascular grafts. For this purpose, discs measuring 15 × 15 × 1 mm were printed using the same conditions as the vascular grafts. MiniFlex II Dekstop Powder X-ray diffractometer (Rigaku Corporation, Kent, UK) bearing Cu Kβ radiation was utilised in this analysis. The scanning was carried out in continuous mode for 2.0°/min at room temperature with a sample width of 0.03° and an angular range of 5–60° 2θ (2 theta). The voltage was set at 30 kV, and the current employed was 15 mA.

#### Mechanical properties

A TA.XTplus texture analyser (Stable Micro Systems, Surrey, UK) was used to assess the mechanical characteristics of the 3D-printed materials. Therefore, strips measuring 50 × 3 × 1 mm were printed with the exact parameters as the vascular grafts. The TA.XTplus texture analyser was set at tension mode, and the strips were stretched between two clamps which were 2 cm apart with a constant extension speed of 10.2 mm/min, and with the maximum stretch distance reaching 20 cm. All measurements were collected using a setup previously described by [27]. After subjecting the strips to stretching, the elastic modulus was calculated by using the slope of the stress versus strain curve [42]. In addition, the maximum possible tensile stress and strain at failure were determined [43].

### Drug release study

#### In vitrorelease study

The release study was carried out using PBS (pH 7.4) with 0.1% w/v Tween 80 under sink conditions. Each graft was weighed and placed in a falcon tube containing 10 mL of the release medium and kept in a rotating incubator (37 °C, 40 rpm). The release study was carried out over the course of 30 days, during which samples were taken daily for the first week and then every 4 days afterwards. At these time points, all the media was swapped out with new media. All the experiments were carried out in triplicates. The concentration of the released CLOP in the media was then determined using the RP-HPLC method described below. Equation (1) was used to compute the cumulative percentage of the released drug [44].

1 $$\mathrm{Cumulative\;release\;}\left(\mathrm{\%}\right)= \frac{\sum\;{W}_{0-\mathrm{t}}}{{W}_{\mathrm{T}}} \times 100$$

where $\sum {W}_{0-\mathrm{t}}$ is the total amount of CLOP released from the graft from time zero to time *t*.${W}_{\mathrm{T}}$ is the initial CLOP cargo within the graph calculated considering the initial drug loading and graft weight.

##### Chromatographic conditions

Analysis of CLOP samples took place using reversed-phase Agilent 1200 Infinity Quaternary System HPLC using an Xselect® CSH™ C18 column with a 3.5-µm particle size and a 3.0 × 150-mm dimension (Waters Corporation, MA, USA). The best peak shape was achieved using a mobile phase consisting of 70% acetonitrile and 30% water (pH 2.5 ± 0.05) at a UV absorbance of 230 nm. The injection volume was set at 10 µL, and the flow rate was set at 0.5 mL/min. A standard calibration curve was plotted within a concentration range of 0.1–50 µg/mL. This curve was then applied to each of the release samples in order to quantify the amount of CLOP present in each sample.

### Platelet adhesion study

Blood platelet deposition on the 3D-printed sample surface was measured using rabbit platelet-rich plasma (PRP), which was produced by centrifuging the rabbit blood in sodium citrate (Avivasysbio, San Diego, CA, USA) at 1900 rpm for 15 min. Vascular grafts containing either no drug or 5%, 10%, and 20% of CLOP were placed in a 96-well plate. Subsequently, samples were incubated with a 200-µL aliquot of the PRP at 37 °C for 1 h. After the incubation, samples were rinsed three times with PBS (pH 7.4) and fixed with a 2.5% glutaraldehyde solution for 2 h. After washing three times with PBS (pH 7.4), samples were dehydrated through a series of ethanol solutions (70% and 100%) for 15 min at each step. After this dehydration step, the samples were allowed to dry at room temperature for 24 h. Finally, SEM (Hitachi TM3030; Tokyo, Japan) micrographs (*n* ≥ 3) were used to count the adhered blood platelets on the scaffold surface.

### Haemocompatibility study

The haemolysis test was performed using rabbit blood in sodium citrate (Avivasysbio, San Diego, CA, USA) to assess the effect of the 3D-printed samples on red blood cells. The 3D-printed samples were cut into a 2-mm disc using a tissue punch and immersed in 1 mL saline solution (0.9% w/v NaCl solution) at room temperature for 2 h. Then 1 mL of rabbit blood was transferred from the original bottle to a 2 mL Eppendorf tube which was then centrifuged at 2000 g for 5 min, and the supernatant was discarded. The resulting pellet was resuspended in 1 mL of a saline solution. This procedure was repeated 3 times. At the end of this step, resuspend the pellet in either deionised water or saline solution to prepare positive and negative controls (positive control: the resuspended pellet in 1 mL of deionised water, negative control: the resuspended pellet in 1 mL of saline solution). The positive and negative controls were then transferred to a glass vial containing 9 mL of saline solution or water. A disc of 3D-printed sample was placed in a 2-mL Eppendorf tube, and subsequently, 200 µL of negative control was added to the tube. The tubes were incubated in the oven at 37 °C for 1 h. The 3D-printed sample was removed from the tube, and then the tube was centrifuged at 2000 g for 5 min. The supernatant was carefully collected and transferred into a 96-well plate to measure the absorbance at 545 nm using an UV/vis spectrometer (FLUOstar Omega Microplate Reader, BMG LABTECH, Ortenberg, Germany). The volume of sample per well was 100 µL. The absorbance of negative and positive controls was also measured for the calculation of the haemolysis percentage using Eq. (2).

2 $$\%\mathrm\;{Haemolysis}=100\times(\frac{\mathrm{AS}-\mathrm{AN}}{\mathrm{AP}-\mathrm{AN}})$$

where AS is the absorbance value of the test sample, AN is the absorbance value of negative control, and AP is the absorbance value of positive control.

### Coagulation test

Three-dimensional–printed samples were cut into rectangles measuring 3 mm × 5 mm × 1 mm and placed in a 2-mL Eppendorf. 0.1 mL of 0.1 M calcium chloride solution was added to the 1 mL of fresh rabbit blood (Avivasysbio, San Diego, CA, USA) and mixed well. The 0.1 M calcium chloride solution was prepared by dissolving 73.5 mg CaCl_2_ dihydrate in 5 mL of water. The 50 µL of blood-CaCl_2_ solution was added to the Eppendorf containing the samples and incubated for 30 and 45 min. Then, 1.5 mL of water was added to each Eppendorf and agitate the Eppendorf at 70 rpm using the plate shaker for 5 min. The solution was collected and 100 µL transferred to a 96-well plate and measured the absorbance at 545 nm using an UV/vis spectrometer (FLUOstar Omega Microplate Reader, BMG LABTECH, Ortenberg, Germany).

### Huvecs growth

Prior to seeding, scaffolds were sterilised under UV irradiation for a total of 1 h and briefly calibrated with the cell culture medium (EGM-2 media (LONZA 00190860)). To evaluate cellular accommodation and growth, HUVECs (ATCC CRL-1730) were seeded onto the smooth surface of the printed scaffolds at a density of 40,000 cells. To allow direct settlement of cells onto the graft, cells were resuspended in 80 µL of media and seeded in a dome on top of each scaffold. Cells were allowed to attach for a total of 8 h. The attachment was confirmed at 8 h post-seeding prior to supplementation with additional cell medium. Thereafter, cells were cultured for an additional 48 h to assess cell viability and proliferation. The cell culture medium was refreshed every 2 days. Viability and cellular proliferation were assessed through live cell staining with NucBlue® Live ReadyProbes® Reagent (Thermo Fisher Scientific, R37605) and using the CyQUANT™ NF Cell Proliferation Assay (Thermo Fisher Scientific, C35006), respectively.

### Statistical analysis

In order to analyse the findings and determine whether or not there were significant variations between the means of the different data sets, a one-way analysis of variance (ANOVA) was carried out, and, where appropriate, the data were presented in mean ± standard deviation. In every instance, a *p*-value of > 0.05 was used to represent the absence of a statistically significant difference, and a *p*-value < 0.05 was used to indicate statistical significance.

## Results and discussion

### 3D printing and characterisation of vascular grafts containing CLOP

**Fig. 1:**
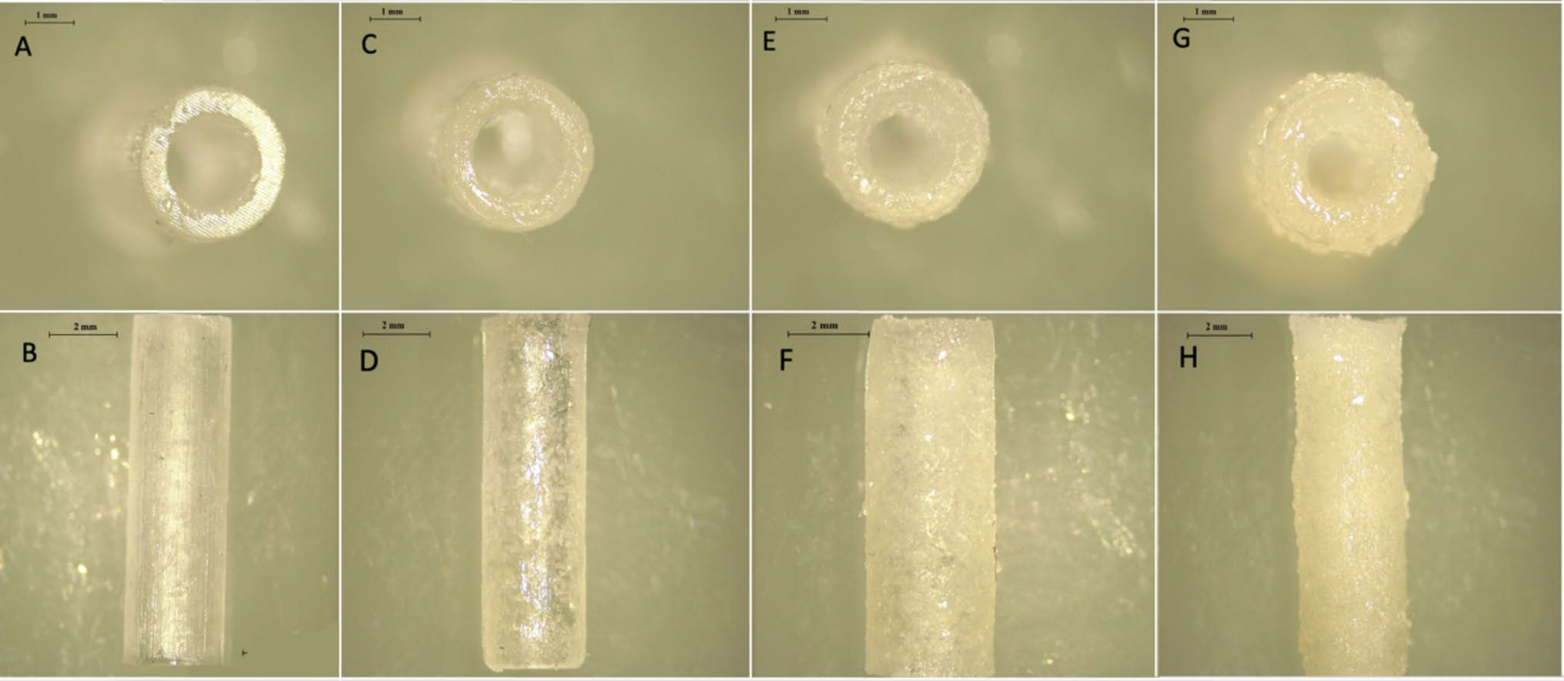
Representative light microscope images of 3D-printed vascular grafts: **A**, **B** blank vascular grafts, **C**, **D** vascular grafts containing 5% w/w CLOP, **E**, **F** vascular grafts containing 10% w/w CLOP, and **G**, **H** vascular grafts containing 20% w/w CLOP. Scale bars: panels (**A**, **C**, **E**, **G**) 1 mm; panels (**B**, **D**, **F**, **H**) 2 mm

CLOP-loaded PLA-PUA/L-PCL-based cardiovascular grafts were developed via 3D printing. Due to the antithrombosis properties of CLOP [45], it may be used to inhibit blood clot development on the surface of biomaterials. Accordingly, four different types of vascular grafts were developed (Fig. 1), including a blank graft (containing no drug) and three types of CLOP-loaded grafts containing 5% w/w, 10% w/w, and 20% w/w of drug. According to prior research conducted by Stewart et al. (2021), the use of L-PCL in combination with other polymers, such as PLA, may speed up the rate of degradation of the implantable devices that are manufactured [46–49]. Furthermore, L-PCL enabled a more effective integration of CLOP into the highly viscous PLA-PUA matrix without the need for solvents (Fig. 1), as these are necessary when employing the electrospinning technique [50]. Indeed, it is possible for humans to be harmed by some types of solvents, in particular when they are used for implanted devices such as vascular grafts [15].

#### Microscope study

Light microscope images of the blank vascular grafts, along with three other types of CLOP-loaded vascular grafts that contained 5% w/w, 10% w/w, and 20% w/w, are shown in Fig. 1. No evidence of drug aggregation was seen on the surface of the 3D-printed grafts containing low drug concentration (5% w/w). However, drug crystals were observed on the graft surface for higher drug loadings, i.e., 10 and 20% w/w. Despite this, the drug was distributed homogenously throughout the manufactured CLOP-loaded vascular grafts. Moreover, the increased white-colour intensity of the grafts is providing a visual evidence of the higher drug loadings.

**Fig. 2:**
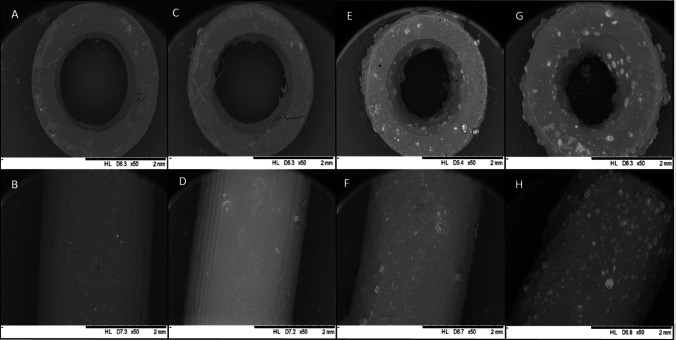
SEM images of vascular grafts and their cross-section containing different drug concentrations. **A**, **B** Blank vascular grafts, **C**, **D** vascular grafts containing 5% w/w CLOP, **E**, **F** vascular grafts containing 10% w/w CLOP, **G**, **H** vascular grafts containing 20% w/w CLOP. Scale bar 2 mm

SEM was used to analyse the surface morphology and assess how the drug was distributed throughout the 3D-printed vascular grafts (Fig. 2), thus complementing the results obtained by using a light microscope (Fig. 1).

Blank vascular grafts had a smooth surface (Fig. 2). Alternatively, CLOP-loaded vascular grafts showed a higher surface roughness. However, this surface roughness followed a clear trend according to the concentration of CLOP available in the vascular grafts, i.e., the surface of 5% CLOP-loaded vascular grafts was closer to the blank vascular grafts rather than the higher drug loadings (10% and 20% w/w). As has been described in the literature, the drug concentration may have an effect on the surface morphology [24, 25, 51]. Moreover, cross-section images are not only corroborating the homogenous distribution of the CLOP throughout the vascular grafts but also show those drug crystals observed on the outer surface of the 3D-printed materials. Figure 2D and F illustrate the surface of the grafts with 5% w/w and 10% w/w CLOP concentration. The drug deposition on the surface of these two drug loadings is not as obvious compared to the graft with the highest drug cargo. However, the drug crystals can be seen clearly in the cross-section images for vascular grafts containing 10% w/w (Fig. 2).

On the other hand, from the higher magnification images of vascular grafts containing greater CLOP-loading (20% w/w), it can be understood that the CLOP is present in crystalline form, which remained the same in comparison to the CLOP pure powder.

#### FTIR spectroscopy

**Fig. 3:**
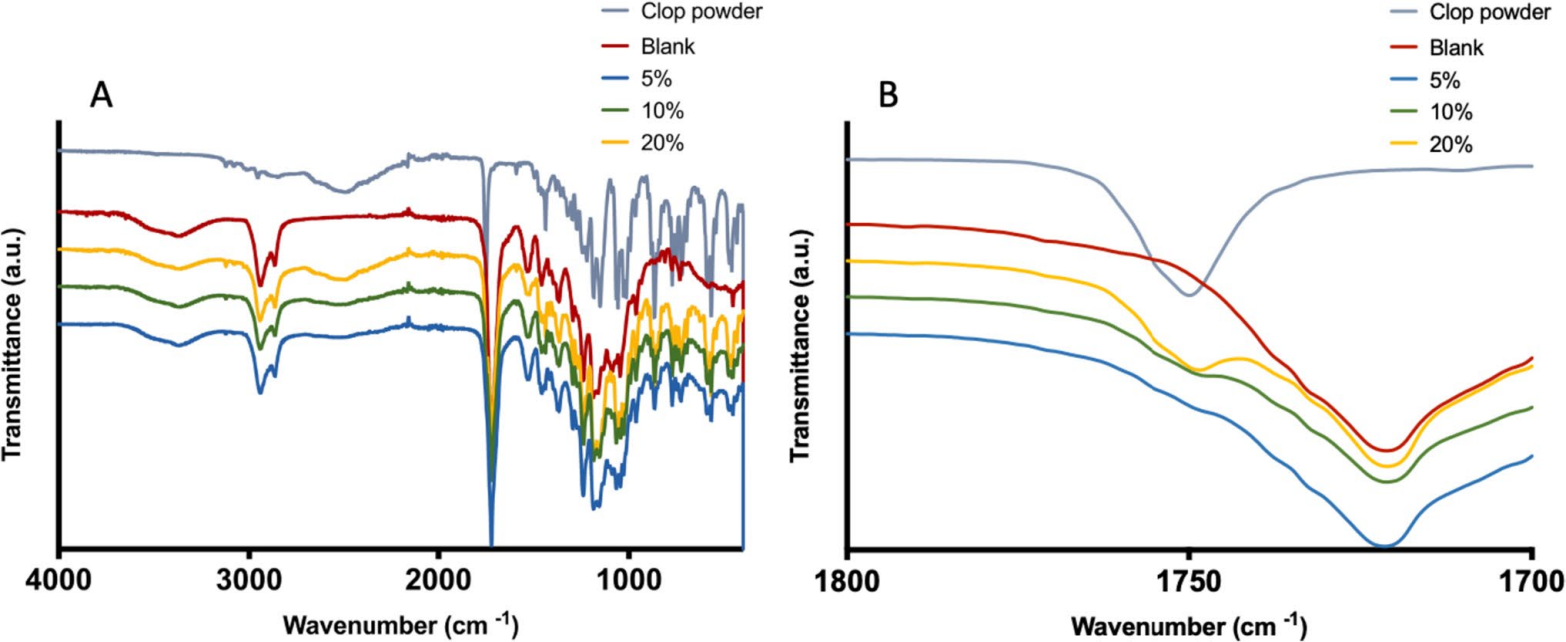
FTIR spectra of CLOP powder and 3D-printed vascular grafts containing different drug loadings (**A**); magnified carbonyl peak of 3D-printed grafts (**B**)

An FTIR analysis was carried out in order to study possible drug-polymer interactions within resulting 3D-printed vascular grafts. The FTIR spectra obtained from the different vascular graft formulations and the pristine CLOP powder are represented in Fig. 3A. The FTIR spectrum of pure CLOP powder is showing a strong characteristic peak at 1752 cm^−1^ owing to C = O stretching vibrations. Moreover, an O–H stretching band was observed at 3012 cm^−1^ due to the hydrogen sulphate moiety. Additionally, the band at 3121 cm^−1^ is due to the stretching vibrations of aromatic C–H bonds, as previously reported in the literature [52, 53]. On the other hand, the FTIR spectra of blank vascular grafts indicated distinctive peaks at 2934 cm^−1^, 2868 cm^−1^, which are attributed to C–H stretching, and 1724 cm^−1^, which is attributed to the C = O stretching [54].

It is interesting to note the characteristic peak of the pure drug was observed in the spectra of the CLOP-loaded 3D-printed vascular grafts. The intensity of these peaks is proportional to the drug cargo of the vascular grafts. Moreover, the carbonyl peak for CLOP (ca. 1750 cm − 1) in the vascular grafts shows a slightly lower wavenumber than the pure CLOP. This is consistent with H-bond interactions between CLOP and the polymers in the vascular graft [55]. In addition, it seems that no chemical reactions occurred between the drug and the polymers during the 3D printing procedure since no new peaks were observed.

#### Thermal analysis

**Fig. 4:**
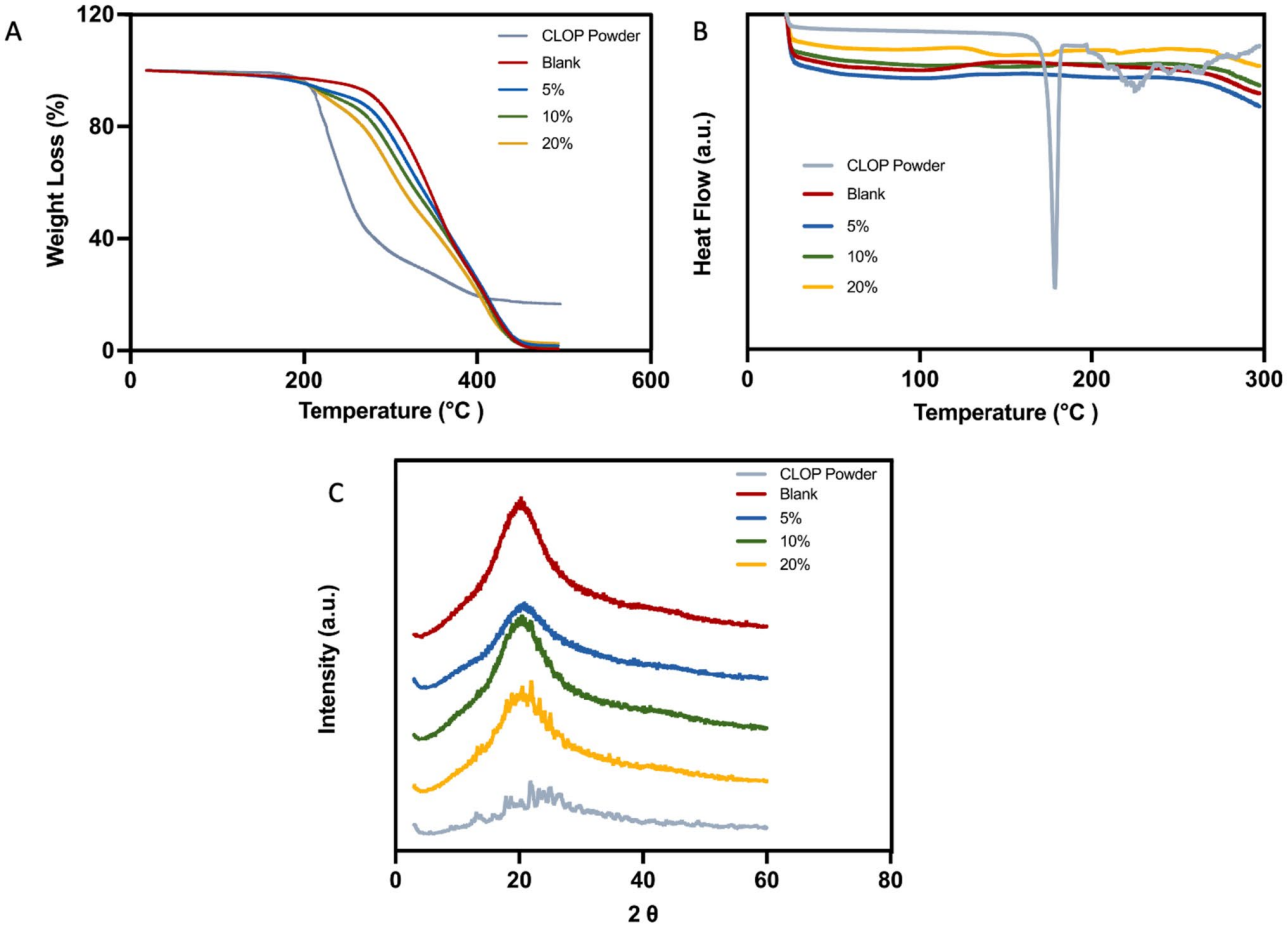
DSC analysis of 3D printing PLA-PUA/L-PCL-based vascular grafts containing different CLOP loadings and CLOP powder (Exo up) (**A**), TGA thermograms of CLOP powder and 3D-printed PLA-PUA/L-PCL-based vascular graft containing different CLOP loadings (**B**). XRD diffractograms of pure CLOP powder and 3D-printed materials containing different CLOP loadings (**C**)

Thermal analysis was used to try to establish if there was any interaction between the polymer and CLOP. TGA analysis (Fig. 4A) showed that when CLOP was combined with the PLA-PUA/L-PCL-based matrix by using 3D printing technology, the resulting material had a different thermal degradation behaviour in comparison with the ones containing no CLOP. Indeed, the *T*_onset_ of the blank grafts was 286 °C, while the *T*_onset_ of the CLOP-loaded vascular grafts was decreasing as the concentration of CLOP was increasing up to 20%. Moreover, the lowest *T*_onset_ was recorded for CLOP powder (209 °C), thus being the drug more thermolabile than the polymer. Similar results have also been described for different drug-loaded polymeric-based matrix [49, 56, 57].

To further investigate these interactions between PLA-PUA/L-PCL and CLOP, DSC analysis was also performed (Fig. 4B). The CLOP DSC curve displayed a characteristic endothermic melting point at a temperature of 180 °C, confirming the drug’s crystalline nature. As expected, this peak was not found in the blank vascular grafts, but neither in the ones that included 5% and 10% w/w of CLOP. Moreover, the DSC thermogram of the 3D-printed vascular graft containing 20% w/w of CLOP showed a very small endothermic peak at 180 °C, which coincides with the melting point of the drug. These results are thus indicating that the formulation of PLA-PUA/L-PCL-based CLOP-loaded vascular grafts considerably reduced the drug crystallinity, which correlated with improved drug solubility. Similar findings have been reported for different polymeric drug-loaded formulations [48, 58–60].

As previously shown in Fig. 2, CLOP particles were found in the SEM images for grafts containing 10 and 20% CLOP; thus, it can be assumed that the amount of crystalline drug is too small to be detected using DSC [59]. Accordingly, an XRD analysis was performed to further determine CLOP crystallinity.

#### XRD analysis

XRD diffractograms of pure CLOP, blank PLA-PUA/L-PCL, and CLOP-loaded PLA-PUA/L-PCL 3D-printed samples are presented in Fig. 4C. CLOP crystallinity when formulated into a 3D-printed vascular grafts implant was evaluated by comparing the peaks observed on their diffractograms with the peaks detected on the diffractogram of pure CLOP. Many distinct peaks in the diffractogram of CLOP were observed at the 2θ diffraction angles of 9.15, 13.44, 14.07, 18.57, 18.69, 22.54, 23.82, and 26.79, indicating that CLOP is presented as a crystalline form. However, the XRD diffractograms of the blank 3D-printed samples showed no diffraction peaks, indicating that the polymeric matrix has an amorphous structure. Interestingly, the diffraction pattern of pure CLOP powder was not found in the diffractograms of the samples containing 5% w/w CLOP cargo. This is presumably due to the result of the interaction between CLOP and PLA-PUA/L-PCL matrix, which lowers the crystallinity of CLOP in the formulation and, as a result, indicates the transformation from a crystalline to an amorphous state and also a uniform distribution of the drug within the polymer. On the other hand, it seems that CLOP exhibits some degree of crystallinity in samples containing 20% w/w and 10% w/w of the drug. This is quite clear in the highest drug loading (20% w/w) of the samples, and it is possible to verify it for the samples containing 10% w/w CLOP due to the existence of minor CLOP peaks at around 22°.

Based on the obtained results, it can be understood that the majority of the CLOP may have been dissolved in the polymer matrix to produce amorphous dispersions, while only a small amount of the drug could have remained in crystalline form. Shitole et al. [61] have reported similar results for CLOP in polyurethane/polyethylene glycol matrix. These findings, therefore, provide additional evidence to support the drug-polymer interactions that were established before by SEM, FTIR, and thermal analysis.

#### Mechanical properties

**Fig. 5:**
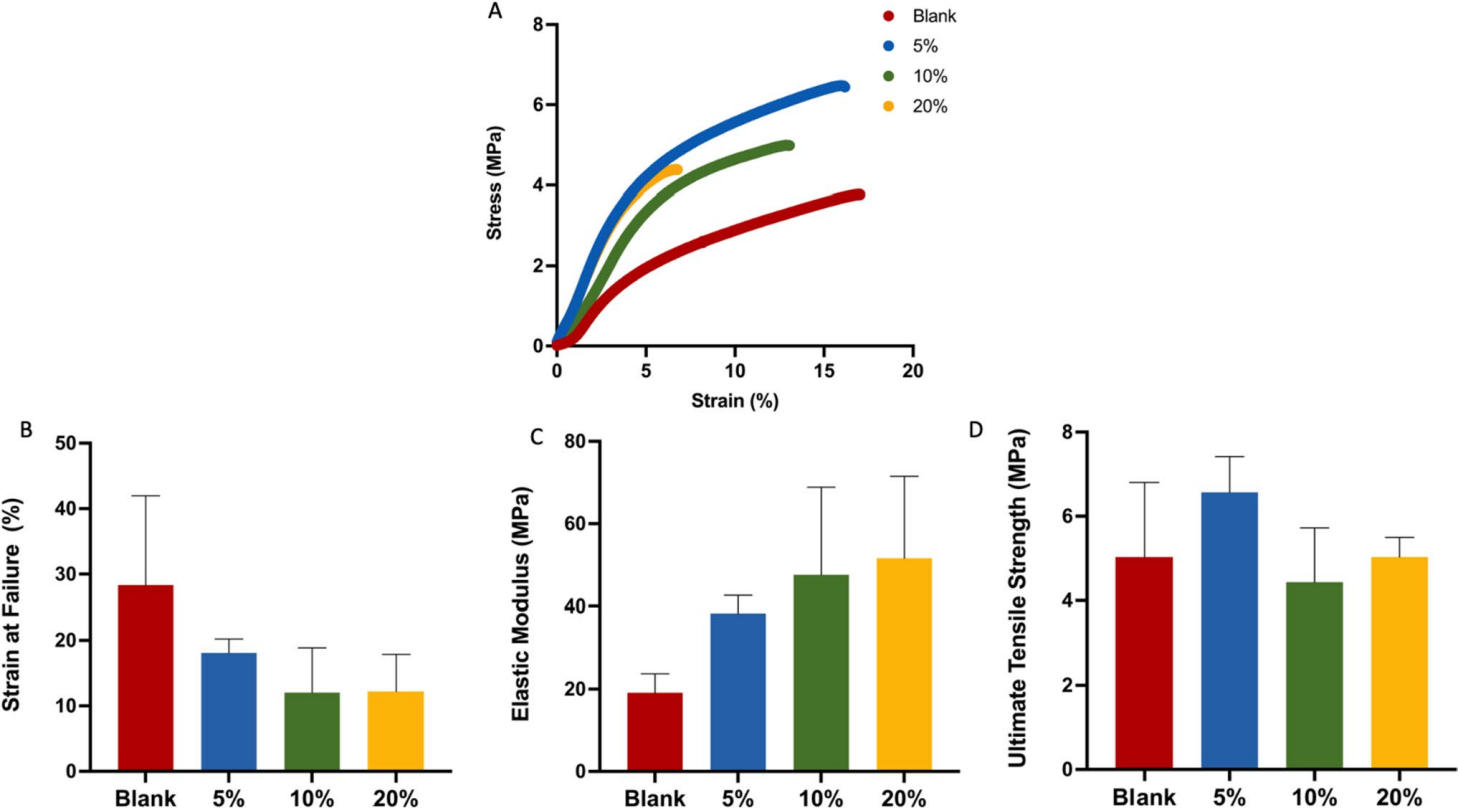
**A** Representative stress/strain curves for the CLOP-loaded 3D-printed materials, **B** strength at failure, **C** elastic modulus,** D** ultimate tensile strength, (means ± SD, n = 3)

The 3D-printed samples were subjected to tensile mechanical testing (Fig. 5A). According to the obtained values for the strain at failure, materials composed of only PLA-PUA/L-PCL (blank) had a higher elasticity (Fig. 5B). Nevertheless, in comparison to the other samples, the samples that contained 20% CLOP w/w demonstrated the lowest degree of strain. However, this difference was not significant. Moreover, the elasticity of these materials falls within the range of the common materials employed for the production of synthetic vascular grafts or natural blood vessels [62, 63]. The strain at failure of the blank materials is higher than the values obtained for the CLOP cargo materials. Nevertheless, these outcomes resemble those attained for human blood vessels (10–105%) [64].

Considering the elastic modulus results, it can be understood that the addition of the CLOP to the polymeric PLA-PUA/L-PCL matrix render stiffer materials than pristine PLA-PUA/L-PCL-based materials (Fig. 5C). It is interesting to note that 3D-printed materials that were developed containing 20% w/w CLOP demonstrated greater stiffness than the other prepared samples. This implies that the material’s stiffness is improved by the presence of CLOP at the higher concentration (20% w/w). It can be mentioned that the elastic modulus values increased as CLOP cargo was increased. This is in line with earlier findings detailing possible interactions between the drug and the polymer matrix discovered through thermal analysis and XRD [26, 28]. Native blood vessels have an elastic modulus of 0.3–1.5 MPa [65], which is comparatively lower than the elastic modulus of the materials obtained in this study. However, in comparison to the elastic modulus of synthetic polymers, which are clinically used to create vascular grafts, such as Dacron (14,000 MPa) or poly(tetrafluoroethylene) (500 MPa), the 3D-printed PLA-PUA/L-PCL-based composites had a considerably lower elastic modulus. Furthermore, the elastic modulus of the PLA-PUA/L-PCL materials is lower than PLA and PCL alone, which have an elastic modulus of 350 MPa and 1–4 GPa, respectively [65].

The ultimate tensile strength of the obtained 3D-printed materials was also measured, and the results are presented in Fig. 5D. The materials containing 5% w/w CLOP had a higher tensile strain in comparison to the other two CLOP loadings (10% w/w, 20% w/w), as well as the blank materials. However, this difference is not significant across all the four formulations. On the other hand, compared to poly(ethylene terephthalate) (170–180 MPa) or poly(tetrafluoroethylene) (14 MPa), the ultimate tensile strength of the obtained 3D-printed materials was considerably lower [65]. However, similar results were reported in the literature for PCL-based materials, which contained a different antiplatelet drug, such as dipyridamole [26]. Furthermore, the obtained values corresponded to the tensile strengths of blood vessels (1.4–11.1 MPa) [65].

### Drug release study

**Fig. 6:**
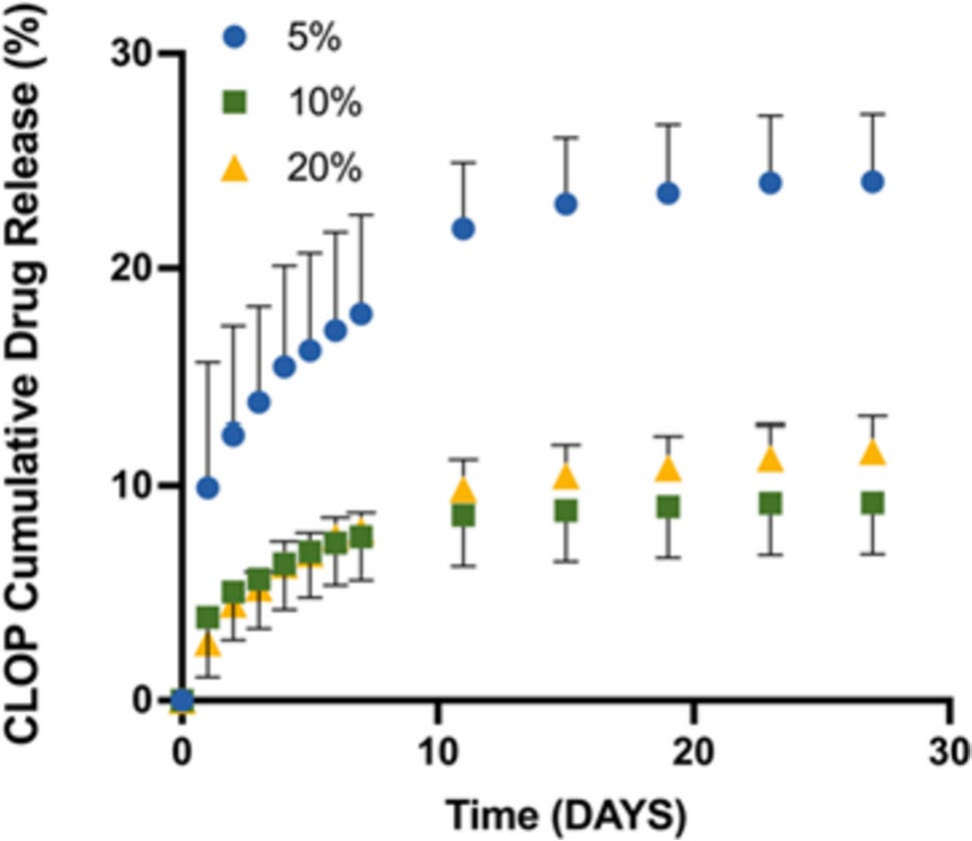
CLOP release from different vascular grafts as a function of time

As mentioned previously, CLOP was loaded in the biomaterials to prevent blood clot formation in the surface of the graft. The anticoagulant activity can be achieved by retaining the drug in the surface of the graft [57, 66]. However, drug released will play a role in the prevention of blood clot formation. Therefore, CLOP release from 3D-printed vascular grafts in PBS (pH 7.4) with 0.1% w/v Tween 80 was assessed over a 27-day period and is illustrated in Fig. 6. An initial burst release was observed for 5% w/w and 10% w/w CLOP-loaded vascular grafts within the first 24 h, which was extended to 48 h for samples containing higher CLOP cargo (20% w/w).

Three-dimensional–printed vascular grafts containing 20% w/w CLOP had a faster release in the first 10 days, but after that, the release slowed to a steady pace that lasted up to 27 days. Meanwhile, these two zones were not seen in vascular grafts with 5% w/w and 10% w/w CLOP. Moreover, after 27 days, there was a significant increase in drug release proportionate to a rise in drug cargo from 10 to 20% (*p* < 0.05), but no such increase was observed with a rise in drug loading from 5 to 10% (*p* > 0.05). The maximum drug release was 1.04 mg which accounts for 11.62% of CLOP with respect to the initial drug loading (Fig. 6) and was seen in the vascular grafts containing 20% w/w CLOP, which may be due to CLOP being more readily available and diffusing out more easily than in vascular grafts with lower drug loadings (5% and 10% w/w). Furthermore, the release pattern shows that the drug-loaded vascular grafts containing 20% w/w of the drug maintain an increasing release rate. In contrast, the 10% w/w and 5% w/w drug-loaded vascular grafts have reached a plateau state after 10–11 days. While one can postulate that the CLOP could interact with the PLA-PUA/L-PCL matrix that is included inside the 3D-printed grafts, inhibiting a greater amount of drug release, this holds true in light of the findings that were discussed in the prior sections. It is important to highlight that the total amount of drug released from grafts containing 5% CLOP is slightly higher than the amount of drug released from grafts containing 10% CLOP. This could be potentially explained by considering previous results regarding drug crystallinity. It has been extensively reported that a more stable drug crystalline form presents a reduced driving force for dissolution when compared with an amorphous drug [67]. Grafts containing 5% CLOP showed a lower degree of drug crystallinity than grafts containing 10% CLOP. Therefore, the drug in grafts containing 5% CLOP will have a faster dissolution profile. Additionally, the results obtained here differ from those reported for other vascular grafts described in the literature loaded with CLOP [61]. These grafts were prepared using electrospinning rather than 3D printing, presenting a high surface area that allows more efficient solvent access and higher drug release. Moreover, as the drug is dissolved with the polymers prior to the production of the grafts, it was in an amorphous form. However, the overall method requires the use of organic solvents, which could pose toxicity issues. Finally, it is worth mentioning that the amount of drug that persists on the surface of the grafts is what really counts in the case of vascular grafts since the surface is in direct contact with the blood and the presence of an antithrombotic drug on the surface helps in the prevention of blood clot formation.

### Platelet adhesion study

**Fig. 7:**
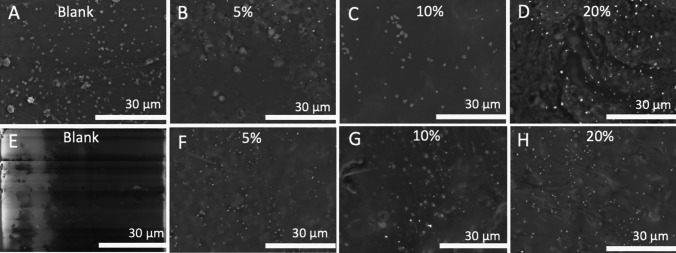
SEM images of vascular grafts surface representing platelet adherence. **A**–**D** The plasma-treated samples and **E**–**H** the non-plasma-treated samples. All the images were taken under 2K magnification

Assessing how blood platelets react to different biomaterials is one of the most prevalent techniques for determining how blood and biomaterials interact with one another. It is widely acknowledged that platelets may play a crucial role in the initiation and progression of the coagulation cascade when blood interacts with a foreign material surface. This cascade has the potential to end up forming a fibrin clot [28, 68]. Therefore, platelet deposition on the surface of the vascular grafts was evaluated using rabbit PRP. Figure 7 shows representative SEM images adhered to the surface of the blank and CLOP-loaded 3D-printed samples. The outcomes demonstrated a substantial reduction in platelet deposition across all CLOP-loaded materials compared to the blank materials without the drug. In brief, no platelets were observed on samples containing 5, 10, and 20% w/w CLOP, while plenty of platelets were deposited on the surface of the blank materials, as can be observed in Fig. 7. The results indicate that the existence of CLOP hinders the attachment of platelets to the surface of the vascular grafts. Furthermore, the reduction in the number of adhered platelets in the CLOP-loaded 3D-printed materials are in line with the reported data on electrospun scaffolds made of PCL and biodegradable elastic polyurethane, loaded with aspirin and dipyridamole, respectively [24, 28].

### Coagulation assessment

**Fig. 8:**
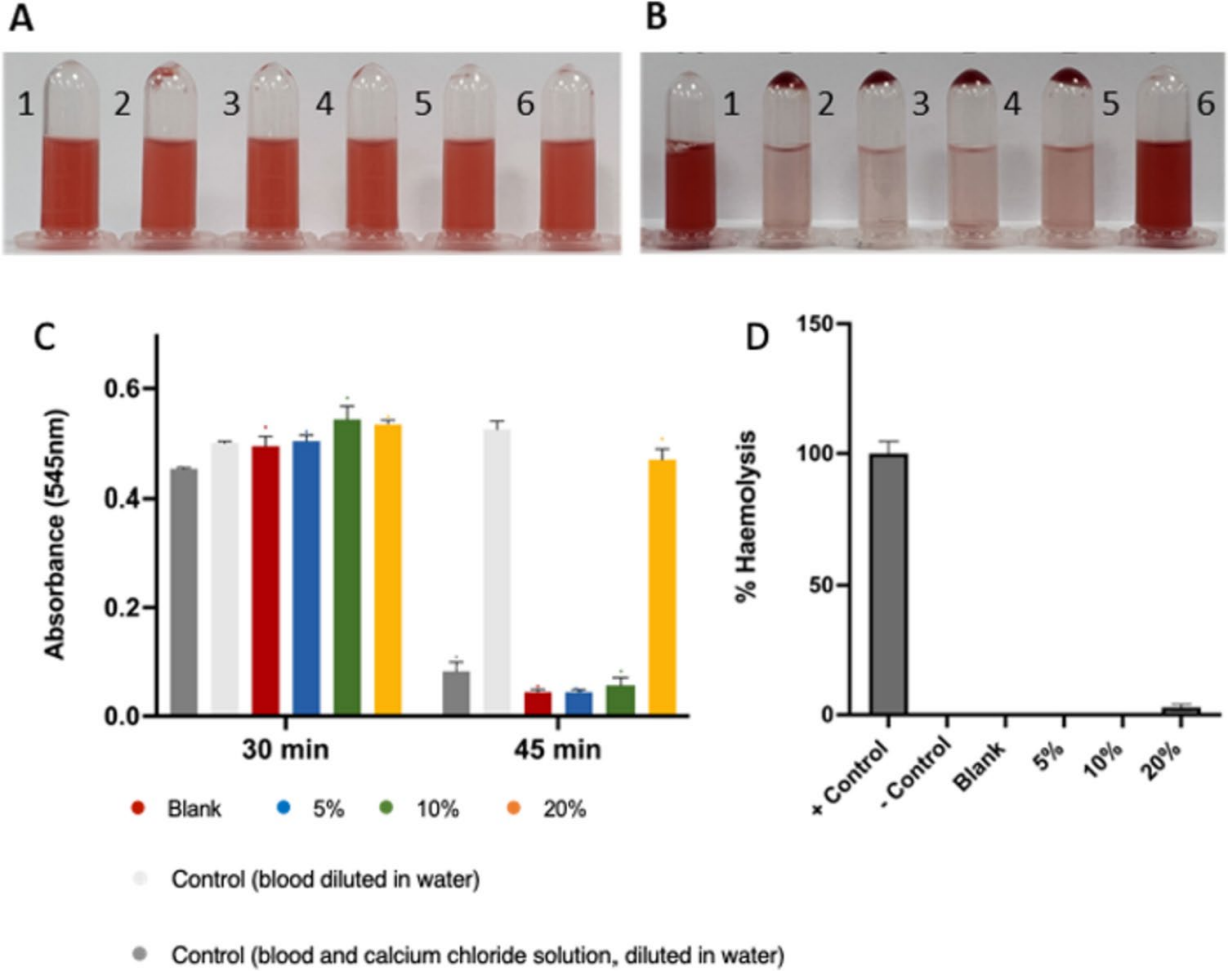
Blood coagulation experiment at **A** 45-min time point and **B** 30-min time point were 1: 50 µL blood and 1.5 mL water, 2: 50 µL blood, 5 µL CaCl_2_, and 1.5 mL water, 3: 50 µL blood, 5 µL CaCl_2_, 1.5 mL water, and blank sample, 4: 50 µL blood, 5 µL CaCl_2_, 1.5 mL water, and 5% drug sample, 5: 50 µL blood, 5 µL CaCl_2_, 1.5 mL water, and 10% drug sample, 6: 50 µL blood, 5 µL CaCl_2_, 1.5 mL water, and 20% drug sample. **C** Coagulation test results displaying absorbance of a solution containing blood at 545 nm for different samples after 30 and 45 min of incubation. **D** Haemocompatibility of CLOP containing 3D-printed samples, blank, and the positive and the negative controls

In order to assess the thrombogenicity of the materials used for the manufacture of implantable devices, a coagulation test was performed [69]. Clot development was evaluated at two different time intervals to determine the required time for a blood clot to form. Figure 8A demonstrates that rabbit blood did not clot on its own during the first 30 min; however, a minor clot was found for the lower drug loadings (5 and 10% w/w) after 30 min. Nevertheless, a full clot was found for the same samples after 45 min (Fig. 8B). Interestingly, no blood clot was obtained after the longer incubation time tested (45 min) by using the sample with the highest drug loading (20% w/w). Therefore, these results clearly indicate that the presence of CLOP in higher concentrations can prevent blood clot formation. Moreover, the absorbance results at 545 nm for all the blood solutions are illustrated in Fig. 8C.

### Haemocompatibility study

**Fig. 9:**
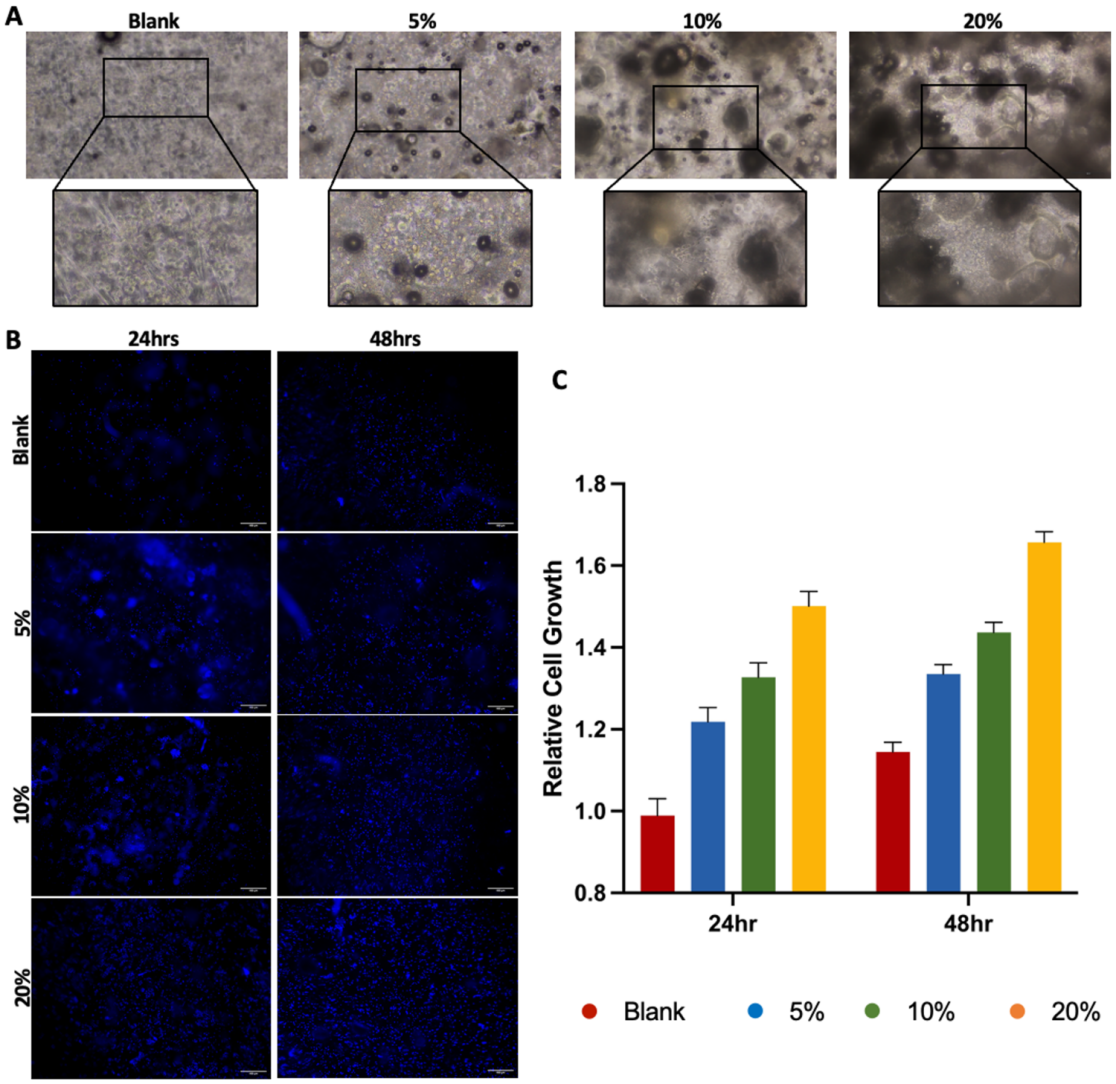
In vitro cytocompatibility, HUVEC attachment on the surface of the blank, 5%, 10%, and 20% loaded materials 8 h post seeding. Scale bar = 200 µm (**A**). Live staining with NucBlue Stain showed HUVEC viability on the surfaces of all samples for up to 48 h following the attachment period. Scale bar = 500 µm (**B**). Following the attachment period, HUVEC growth was assessed using a CyQUANTTM NF Cell Proliferation Assay for up to a total of 48 h. (n = 3)

The manufactured materials developed in this study are intended to be used as vascular grafts. Thus, the haemocompatibility assay of 3D-printed products was performed in order to assess the compatibility of the 3D-printed materials with blood. The results of the haemocompatibility assay are presented in Fig. 9. Although the 3D-printed samples containing the highest drug loading (20% of CLOP) showed the highest haemolysis percentage (3.4 ± 0.7), haemolysis levels were below 5% in all the samples. According to the literature, samples are considered to be haemocompatible if they exhibit less than 5% haemolysis [70].

### Huvecs growth

The biocompatibility of the drug-loaded grafts was determined by culturing HUVECs onto the different samples for a total of 72 h. As can be seen in Fig. 9A, despite the different compositions, cells readily attached to all grafts within the 8-h initial attachment period, with no noticeable differences between scaffolds. To evaluate the ability of the scaffolds to support cell adherence, viability, and growth, scaffolds were evaluated at 24 and 48 h post the attachment period. Live staining with NucBlue® Live ReadyProbes®, a live nuclear stain that fluoresces blue when bound to DNA, confirmed the attached cells were functional and alive and the grafts to be non-cytotoxic (Fig. 9B). While all graphs supported a vast number of functional cells, both 24- and 48-h cells appeared to densely cover a greater total distance of the grafts proportional to the loaded drug content.

Nevertheless, an observable increase in stained cells was observed at the later time point across all sample types, suggesting the scaffolds provided an opportune environment for cell growth. To further assess whether the scaffolds provided a suitable milieu for cellular accommodation, a CyQUANT NF cell proliferation assay was performed at the two time points. As seen in Fig. 9C, a statistically significant increase in cell growth was detected at both the assessed time points between the blank and drug-loaded grafts, with the highest cell growth being observed in the grafts with the highest drug content. Moreover, at the 48-h time point, aligning with the observed differences, a significant increase in cell growth was observed proportionally with drug content.

## Conclusion

DLP 3D printing technology was used to effectively fabricate PLA-PUA/L-PCL-based small-diameter cardiovascular grafts containing an antithrombotic drug (CLOP). It appeared from the characterisation of the samples that the CLOP was in an amorphous state following the printing procedure. However, a small degree of crystallinity was observed on higher drug loading of vascular grafts (20% w/w). Furthermore, the findings of the thermal analysis provide more evidence that CLOP and the PLA-PUA/L-PCL matrix interact with one another. The 3D-printed samples were assessed for mechanical properties. According to the findings, it appeared that these samples had mechanical characteristics that were equivalent to those of native blood vessels. In addition, the 3D-printed sample with the highest drug loading (20%) was able to provide a steady CLOP release for 27 days with a small burst release during the first 48 h. In addition, the platelet deposition over the surface of all the CLOP-loaded 3D-printed materials was significantly reduced in comparison to the blank materials containing no medication. All 3D-printed samples had haemolysis rates of less than 5%. Furthermore, it should be noted that 3D-printed materials have shown the capability to provide a conducive environment for cellular adhesion, viability, and growth. A significant augmentation in cellular proliferation was seen when comparing the blank grafts to the grafts supplied with the drug.

Considering the results described here, it can be concluded that 5% CLOP grafts could be used to produce well-printed grafts while still retaining antiplatelet activity. Grafts containing higher CLOP loadings exhibited good anticoagulant results, but they had poorer printability, resulting in grafts with highly rough surfaces and a high concentration of crystals on the graft surface. Additionally, a higher drug content led to stiffer materials.

Additive manufacturing has been successfully utilised in the pharmaceutical and biomedical industries for the past decade. However, the recent advancements in 3D printing technology, particularly in the development of implantable devices and oral dosage forms, have mostly remained in the realm of research, with only a few approvals from regulatory bodies like the FDA [71]. Moreover, ensuring the sterility of these products is crucial. They must either be sterile prior to use or manufactured in a sterile environment. Consequently, further investigation is required to address the sterility aspect of 3D printing technology. Similarly, the establishment and enhancement of point-of-care (PoC) facilities could facilitate the integration of 3D printing technology into patient care centres, such as hospitals, while ensuring compliance with relevant regulatory standards, ultimately benefiting the patients.

## Data Availability

The datasets generated during and/or analysed during the current study are available from the corresponding author on reasonable request.
